# Supplementary material for: Nitric Oxide and Globin Glb1 Regulate Fusarium oxysporum Infection of Arabidopsis thaliana
Source: Antioxidants (Basel). 2023 Jun 21;12(7):1321. doi: 10.3390/antiox12071321 (PMC10376111; doi:10.3390/antiox12071321)
Supplement: Supplementary file 1 [file antioxidants-12-01321-s001.zip › antioxidants-2407854-supplementary.pdf]

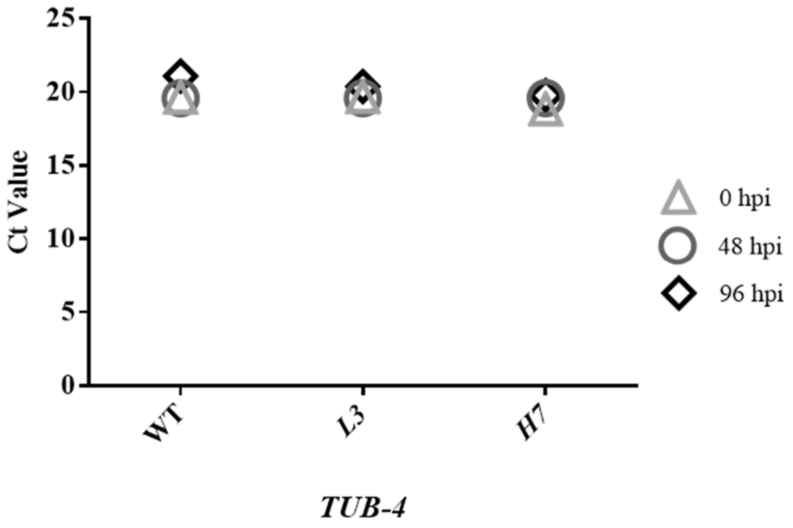

Supplemental Figure S1. *TUB4* expression stability under our experimental conditions. Mean of RT-qPCR Ct values of the selected candidate as a reference gene (*TUB4*) in WT, *L3* and *H7* Arabidopsis seedlings under control treatment (0 hpi) and *F. oxysporum* infection (48 and 96 hpi).

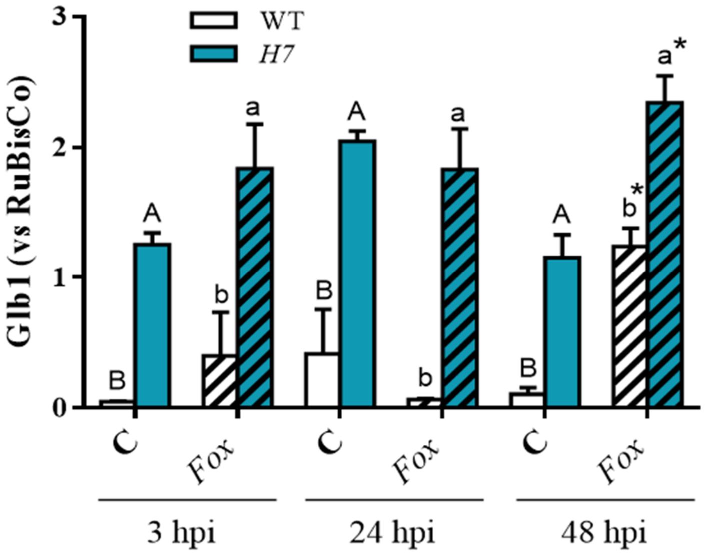

Supplemental Figure S2. GLB1 content in Arabidopsis roots in response to *F. oxysporum.* Western-blot quantification of GLB1 in WT and *H7* Arabidopsis roots at 3, 24 and 48 hpi infected or not (C) with *F. oxysporum* relatively expressed *vs* Ponceau bands. Data represent the mean ± SEM of at least 2 independent experiments. Different letters denote significant differences between genotypes (capital letters in control conditions and lowercase under infection conditions) according to Tukey´s multiple comparison test ($P < 0.05$). Asterisks denote significant differences respect to control within each genotype, in each time point according to T-Student test ($P < 0.05$).

(A)

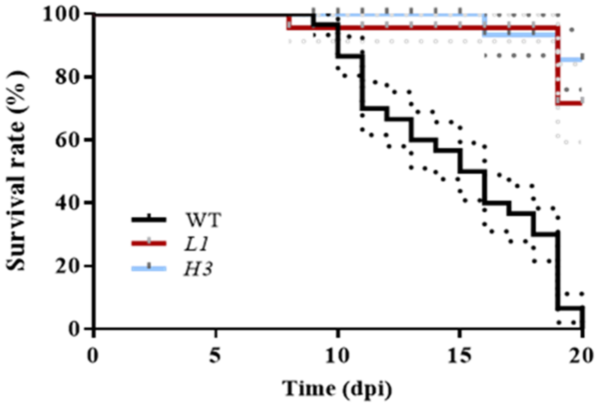

(B)

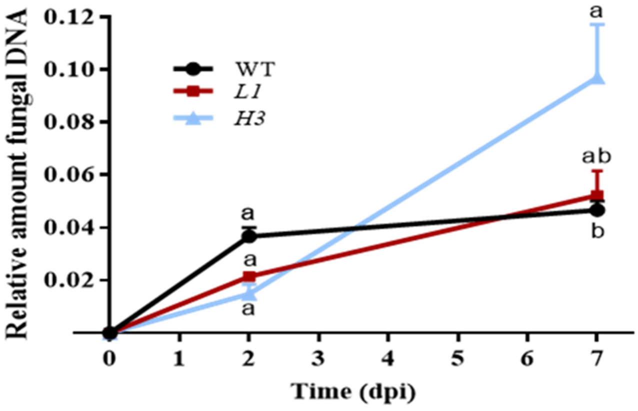

Supplemental Figure S3. Disease development after *F. oxysporum* inoculation of *Glb1* mutants: *L1* and *H3*. (A) Kaplan-Meier plot of the WT, *L1* and *H3* Arabidopsis seedlings survival infected with *F. oxysporum* over the course of 20 dpi. (B) Fungal burden at 0, 2 and 7 dpi determined by RT-qPCR analysis of the *F. oxysporum Actin* gene relative to the Arabidopsis *TUB4* gene. Data represent the mean ± SEM of at least 3 independent experiments. There´s no significant differences in (B) between genotypes in none of the time points according to Tukey´s multiple comparison test (P < 0.05). Asterisks in (B) denote significant differences respect to control (0 hpi) according to Dunnett´s multiple comparison test (P < 0.05).

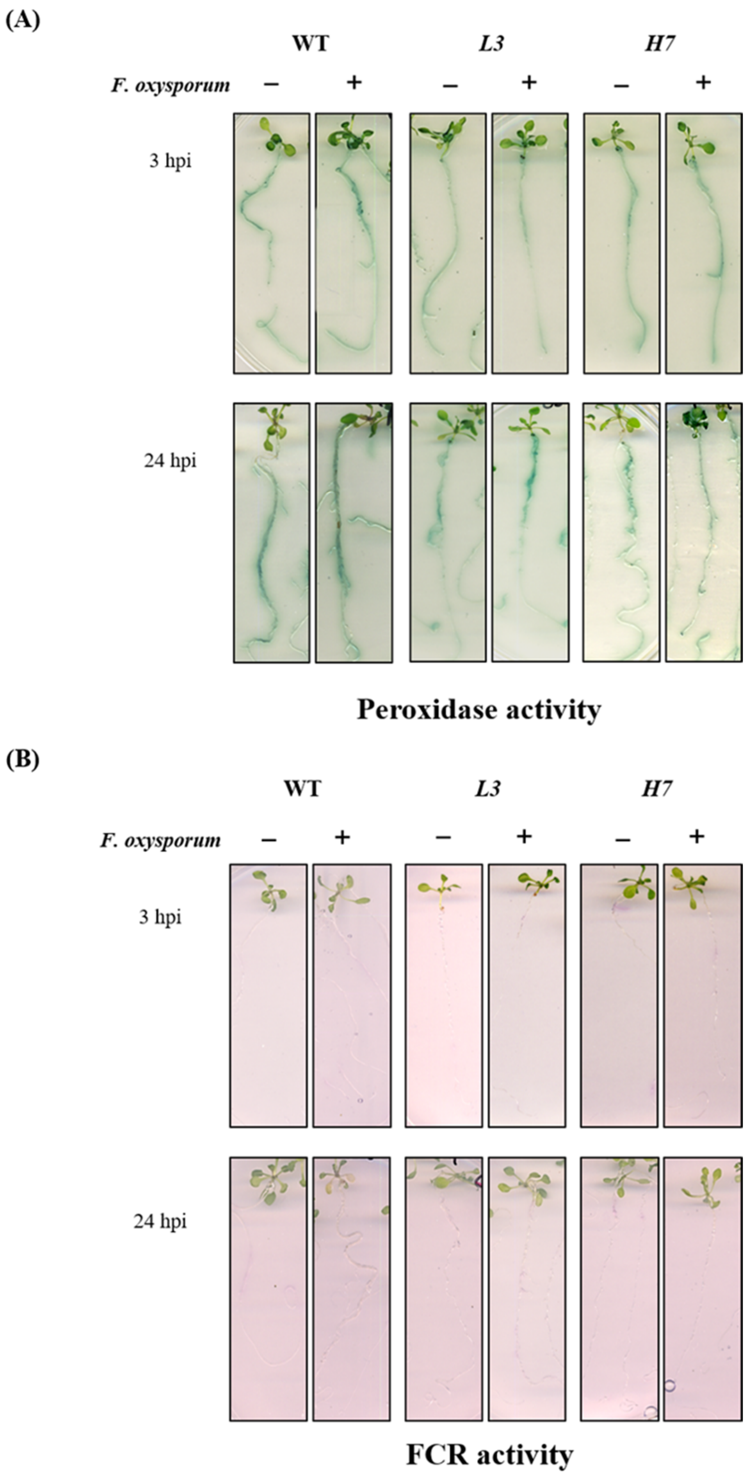

Supplemental Figure S4. Peroxidase and ferric chelate reductase (FCR) activity in Arabidopsis roots after *F. oxysporum* infection. Representative images showing peroxidase (A) or ferric chelate reductase (FCR; B) activity in WT, *L3* and *H7* Arabidopsis seedling roots before (-) and after (+) *F. oxysporum* inoculation (3 and 24 hpi). Images are representative of at least three independent experiments.

Supplemental Table S1. Oligonucleotides used for RT-qPCR

Gene	Primer sequence	ID	T°	Amp	Eff.	References
PDF 1.2-s	AGTTGTGCGAGAAGCCAAGT	AT5G44420	**60**	**107**	**1.98**	(Fernández-Calvo et al., 2011)
PDF 1.2-as	GTTGCATGATCCATGTTTGG					
PR1-s	TCCGCCGTGAACATGTGGGTTAG	AT2G14610	**55**	**180**	**2.01**	Beacon desing
PR1-as	CCCACGAGGATCATAGTTGCAACTGA					
PR5-s	CGGTACAAGTGAAGGTGCTCGTT	AT1G75040	**55**	**312**	**1.94**	Beacon desing
PR5-as	GCCTCGTAGATGGTTACAATGTCA					
FIT-s	ATCCTTCATACGCCCTCTCC	AT2G28160	**60**	**149**	**1.99**	Beacon desing
FIT-as	GAGCCGGTGGTGAAGAAG					
IRT1-s	CGGTTGGACTTCTAAATGC	AT4G19690	**55**	**165**	**1.97**	(Besson-Bard et al., 2009)
IRT1-as	CGATAATCGACATTCCACCG					
Foxc act1-as	ATGTCACCACCTTCAACTCCA	FOXG_04579	**55**	**300**	**2**	(Masachis et al., 2016)
Foxc act1-s	CTCTCGTCGTACTCCTGCTT					

Supplemental Table S2. Reverse transcription quantitative PCR parameters according to the Minimum Information for publication of Quantitative real-time PCR Experiments (MIQE) guidelines derived from Bustin et al., 2009 [48].

**Sample/Template**	
Source	*Arabidopsis thaliana* seedling.
Method of preservation	Harvest in liquid nitrogen, storage at -80 °C.
Storage time (if appropriate)	Maximum one week.
Handling	Frozen.
Extraction method	Trizol reagent (Invitrogen).
RNA: DNA-free	DNA-free™ DNA Removal Kit (Ambion DNA free).
	Use of intron-spanning primers.
	Verification of single peak on dissociation curves (Melting curve).
RNA: concentration	NanoDrop® ND-1000 spectrophotometer/ and agarose (1%) electrophoresis gel.
RNA: integrity	NanoDrop® ND-1000 spectrophotometer and agarose (1%) electrophoresis gel.
**Assay optimisation/validation**	
Accession number	References cited in Materials and methods.
Amplicon details	References cited in Materials and methods.
Primer sequence	References cited in Materials and methods.
In silico	Primer-BLAST (http://www.arabidopsis.org/Blast/index.jsp).
Empirical	Primer concentrations of 250 nM.
	Annealing temperature of 55 or 60 °C.
Priming conditions	Combination of oligo-dT primers and random hexamers.
PCR efficiency	Dilution curves (slope, deviation).
Linear dynamic range	Samples are within the range of the efficiency curve.
**RT and qPCR**	
Protocols	iCycler iQ Real-Time PCR Detection System (Bio-Rad).
	PrimerScript RT reagent Kit (Takara).
	SYBR Premix Ex Taq™ II (Takara) .
	As described in the Materials and methods section.
Reagents	As described in the materials and methods section.
NTC	Cq and dissociation curve verification.
**Data analysis**	
Specialist software	e iCycler Program (Bio Rad).
Statistical justification	As described in the Materials and methods section.
Transparent, validated normalisation	Minimum five references genes selected using the GrayNorm algorithm.
	As described in the Materials and methods section.
